# Mitochondrial genome of *Monolepta hieroglyphica* (Coleoptera: Chrysomeloidea: Chrysomelidae) and phylogenetic analysis

**DOI:** 10.1080/23802359.2021.1914522

**Published:** 2021-04-26

**Authors:** Wei Li, Shuo Shen, Hongyu Chen

**Affiliations:** a Academy of Agriculture and Forestry Sciences, Qinghai University, Xining, China; b State Key Laboratory of Plateau Ecology and Agriculture, Qinghai University, Xining, China; c Key Laboratory of Agricultural Integrated Pest Management of Qinghai Province, Scientific Observing and Experimental Station of Crop Pest in Xining, Ministry of Agriculture, Xining, China

**Keywords:** Galerucinae, pest, mitochondrial genome, phylogenetic analysis

## Abstract

*Monolepta hieroglyphica* (Motschulsky, 1858) is a major pest of potato, maize, cotton and sorghum in China. In this study, we sequenced and analyzed the complete mitochondrial genome (mitogenome) of *M. hieroglyphica*. This mitogenome was 15,761 bp long and encoded 13 protein-coding genes (PCGs), 22 transfer RNA genes (tRNAs) and two ribosomal RNA unit genes (rRNAs). Gene order was conserved and identical to most other previously sequenced Galerucinae Most PCGs of *M. hieroglyphica* have the conventional start codons ATN (six ATT, five ATG and one ATC), with the exception of *nad1* (TTG). Except for three genes (*cox1*, *nad4* and *nad5*) end with the incomplete stop codon T––, all other PCGs terminated with the stop codon TAA or TAG. The whole mitogenome exhibited heavy AT nucleotide bias (80.0%). Phylogenetic analysis positioned *M. hieroglyphica* in a well-supported clade within the subfamily Galerucinae with *Monolepta occifluvis*, *Monolepta* sp. and *Paleosepharia posticata*. These results provided an important basis for further studies on mitochondrial genome and phylogenetics of Galerucinae.

The family Chrysomelidae, commonly known as leaf beetles, includes over 37,000 species in more than 2000 genera, making it one of the largest and most commonly encountered of all beetle families (Thormann et al. 2016). Most leaf beetles are small to medium-sized, with the antennae notably shorter than head, thorax and abdomen. The Galerucinae is the largest subfamily of the Chrysomelidae and includes about 14,500 described species occurring worldwide (Nie et al. 2018). Adult and larval leaf beetles within Galerucinae feed on all sorts of plant tissue, and all species are fully herbivorous (Nie et al. 2018; Zheng et al. 2020). Many of them are serious pests of cultivated plants, for example the widespread leaf beetle *Monolepta hieroglyphica* (Motschulsky, 1858), which can damage potato, maize, cotton and sorghum. Herein, we sequenced and analyzed its mitogenome.

Five male adults of *M. hieroglyphica* were collected from Tianshui City, Gansu Province, China (34°27′N, 105°43′E, July 2019) and were stored in Entomological Museum of Qinghai University (Accession number QHU-EMMH02). Total genomic DNA was extracted from muscle tissues of the thorax using DNeasy DNA Extraction kit (Qiagen, Hilden, Germany). A pair-end sequence library was constructed and sequenced using Illumina HiSeq 2500 platform (Illumina, San Diego, CA), with 150 bp pair-end sequencing method. A total of 20.4 million reads were generated and had been deposited in the NCBI Sequence Read Archive (SRA) with accession number SRR13319950. Raw reads were assembled using MITObim v 1.7 (Hahn et al. 2013). By comparison with the homologous sequences of other Galerucinae species from GenBank, the mitogenome of *M. hieroglyphica* was annotated using software GENEIOUS R11 (Biomatters Ltd., Auckland, New Zealand).

The complete mitogenome of *M. hieroglyphica* is 15,761 bp in length (GenBank accession no. MW419951), and contains the typical set of 13 protein-coding, two rRNA and 22 tRNA genes, and one non-coding AT-rich region. Gene order was conserved and identical to most other previously sequenced Chrysomelidae (Nie et al. 2018; Song et al. 2018; Long et al. 2019; Wang et al. 2020). The nucleotide composition of the mitogenome is 80.0% A + T content (A 41.1%, T 38.9%, C 11.9%, G 8.1%). Four PCGs (*nad4*, *nad4l*, *nad5* and *nad1*) were encoded by the minority strand (N-strand) while the other nine were located on the majority strand (J-strand). The 22 tRNA genes vary from 61 bp (*trnC* and *trnL1*) to 71 bp (*trnV*). Two rRNA genes (*rrnL* and *rrnS*) locate at *trnL1*/*trnV* and *trnV*/control region, respectively. The lengths of *rrnL* and *rrnS* in *M. hieroglyphica* are 1273 and 740 bp respectively, with AT contents of 83.2% and 84.2%, respectively. Most PCGs of *M. hieroglyphica* have the conventional start codons ATN (six ATT, five ATG and one ATC), with the exception of *nad1* (TTG). Except for three genes (*cox1*, *nad4* and *nad5*) end with the incomplete stop codon T––, all other PCGs terminated with the stop codon TAA or TAG. Genetic biodiversity of this worldwide pest in China is still unclear, which can be resolved by sequencing more genetic data of different geographical populations. Compared with the mitogenome of *M. hieroglyphica* which already existed in Genbank (MT192098, collected from Zhoukou City, Henan Province, China) (Li et al. 2020), gene rearrangement, base composition and other general features of this two mitochondrial genomes were similar to each other. However, 96.3% percentage of pairwise identity was shown based on the MAFFT alignment, indicating the obvious nucleotide diversity of *M. hieroglyphica* between this two geographical populations, this may be caused by the geographical isolation and can provide important genetic information for futher population genetic structure analysis of *M. hieroglyphica*.

Phylogenetic analysis was performed based on the nucleotide sequences of 13 PCGs from 23 Coleoptera species. Alignments of individual genes were concatenated using SequenceMatrix 1.7.8 (Vaidya et al. 2011). Optimal nucleotide substitution models and partition strategies were chosen by PartitionFinder v1.1.1 (Lanfear et al. 2012). Following the partition schemes suggested by PartitionFinder, phylogenetic tree was constructed using MrBayes 3.2.6 (Ronquist and Huelsenbeck 2003). Two simultaneous runs with four independent Markov chains were run for five million generations and trees were sampled every 1000th generation. After the average standard deviation of split frequencies fell below 0.01, the first 25% of samples were discarded as burn-in and the remaining trees were used to generate a consensus tree and calculate the posterior probabilities (PP). Phylogenetic analysis positioned *M. hieroglyphica* in a well supported clade within the subfamily Galerucinae with *Monolepta occifluvis*, *Monolepta* sp. and *Paleosepharia posticata* (Figure 1), indicating genus *Monolepta* had a close relationship with *Paleosepharia*. The monophyly of *Monolepta* could not be confirmed by this phylogenetic tree. These results provided an important basis for further studies on mitochondrial genome and phylogenetics of Galerucinae.

**Figure 1.**
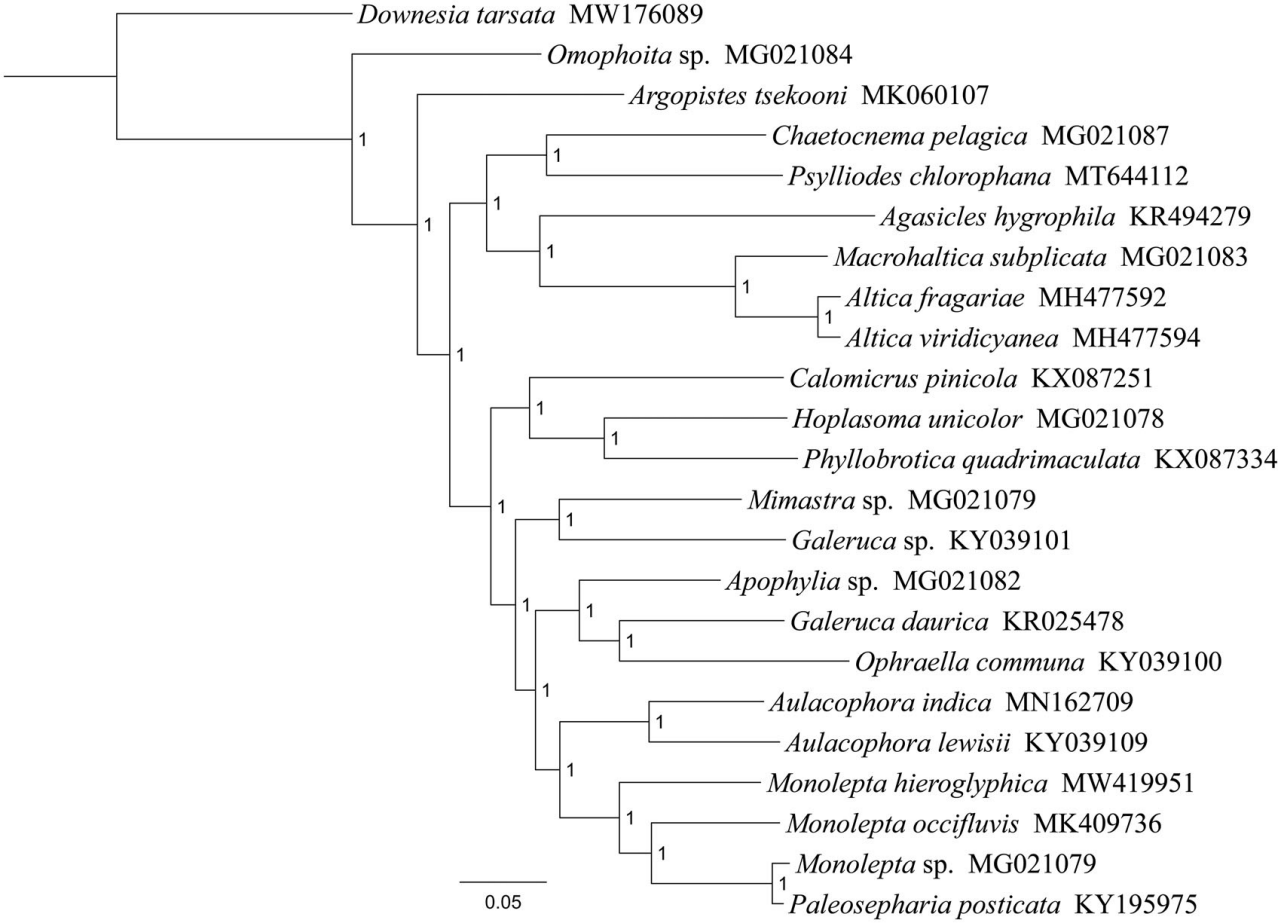
Phylogenetic tree based on the 13 mitochondrial protein-coding genes sequences inferred from Bayesian. Numbers on branches are posterior probabilities (PP).

## Data Availability

The data that support the findings of this study are openly available in NCBI (National Center for Biotechnology Information) at https://www.ncbi.nlm.nih.gov/, reference number MW419951. The associated BioProject, SRA, and Bio-Sample numbers are PRJNA688388, SRR13319950, and SAMN17174216 respectively.
